# The post-COVID-19 pandemic: perspectives of professional practice in nursing

**DOI:** 10.1590/0034-7167.202376Suppl101

**Published:** 2023-10-09

**Authors:** Maria Lucia do Carmo Cruz Robazzi, Fernanda Ludmilla Rossi Rocha, Maria Helena Palucci Marziale

**Affiliations:** I Universidade de São Paulo. Ribeirão Preto, São Paulo, Brazil; II Centro Colaborador da Organização Mundial da Saúde. São Paulo, São Paulo, Brazil; III Universidade Federal de Alfenas. Alfenas, Minas Gerais, Brazil

During the COVID-19 pandemic, nursing workers were essential in providing health care, given the high number of people infected with SARS-CoV-2 viruses and sick with COVID-19 and Severe Acute Respiratory Syndrome (SARS). In this context, health services were overloaded, and many of their professionals fell ill and died.

Nursing proved to be essential during the critical phase of this health emergency, acting directly in care, in the so-called “front line”, drawing attention to its evaluative quality. Its work focused on the recovery and adaptation of patients with functional, motor, psychosocial and spiritual limitations, due to restriction of some actions due to the disease^(1)^.

Months have passed and, after the end of the pandemic decreed by the World Health Organization (WHO) on May 5, 2023^(2)^, this editorial seeks to draw nursing professionals’ attention to what has been evidenced as problems related to the post-pandemic.

How important have nursing workers been in the care stages of COVID-19 survivors?

The analysis of the profile of deaths registered in medicine and nursing professionals, which account for 72.5% of deaths identified among specialized workers who make up the health area in Brazil, showed that the vulnerability identified was a consequence of work overload and precariousness, difficulty in accessing personal protective equipment in the initial months of the pandemic, among other factors related to daily work. Middle-level workers (assistants and technicians) and black workers (blacks and browns) constituted the majority among those killed in nursing teams during the pandemic period^(3)^.

Nursing professionals also directed attention and nursing care to post-COVID-19 patients, presenting problems considered as Post-COVID-19 Syndrome (PCVS-19), defined as new, recurrent or persistent clinical manifestations, present after acute SARS-CoV-2 infection, and not attributed to other causes. In the literature, these clinical manifestations can also be described as long-term COVID, acute post-COVID, long-term effects of COVID, chronic COVID^(4)^.

PCVS-19 was studied in an international panel involving patients, physicians, researchers and WHO staff with the aim of developing a consensus definition for this condition, and the following conclusion was reached: PCVS-19 occurs in individuals with a history of probable or confirmed SARS-CoV-2 infection, usually within three months of onset, with symptoms that last at least two months and cannot be explained by an alternative diagnosis. Common symptoms include fatigue, shortness of breath, and cognitive dysfunction, and patients often experience impacts on their daily performance. Symptoms may be new, following initial recovery from an acute episode of COVID-19, or persist from the initial illness; they can also fluctuate or recur over time. Therefore, changes in daily tasks can occur in most cases^(5)^, which obviously includes work activities.

In addition to these parameters presented by the WHO, studies have been developed involving the post-COVID stage, confirming and/or finding other factors that have been causing fatigue, cardiopulmonary symptoms, physical dysfunctions, cognitive/psychosomatic changes, varied functional limitations, hemostatic sequels, deterioration in quality of life, Diabetes Mellitus, neurological manifestations, among others, inferring that there is, in addition, a vast and unexplored field for carrying out scientific research related to COVID-19.

Thus, with studies still being conducted and multiple approaches to be considered, there is a fruitful field of interest that needs the action of both health professionals in general and nursing workers related to PCVS-19. In other words, the importance of this profession continues to be shown in the care stages of COVID-19 survivors.

There is still much to be done professionally, and nursing’s participation is unequivocal. For instance: nurses specialized in rehabilitation can seek multiple interventions in sequel patients; professionals involved in the care for patients with chronic-degenerative diseases can carry out numerous actions/guidelines for hypertensive, diabetic individuals, with various pains; specialists in hematology nursing can assist in the treatment and rehabilitation of individuals who have acquired sequels; nursing professionals who work in mental health care will certainly be able to alleviate the neuropsychiatric and psychological changes that still affect survivors of this infectious outbreak; those who are specialists in workers’ health will be able to contribute to promoting actions aimed at greater safety in their work environments, aiming to minimize their own illnesses and those of their wards, among others. Moreover, researchers in the profession will continue to investigate other facets of the post-pandemic.

Thus, COVID-19, at its peak of contamination and now, having been considered finished^(2)^, showed nursing workers’ importance in the care stages of infected patients. They ended up disclosing and publicizing all their value and importance, because, despite experiencing insecurity, illness and death in their work environments, they proved to be resilient, overcoming their problems and meeting virtually all of their demands, even increasing their visibility and recognition worldwide.
